# Cesarean Section at 38 Gestational Weeks for Placenta Previa was Associated With Improved Neonatal Outcomes Without an Apparent Increase in Maternal Risks: A Historical Control Study

**DOI:** 10.1155/ogi/1616243

**Published:** 2026-07-24

**Authors:** Minori Takada, Morikazu Miyamoto, Yohei Kaishi, Yuna Tanaka, Akari Imauji, Kimitaka Iwama, Taro Miyake, Kazuya Yoshimoto, Risa Tanabe, Soko Nishimura, Tsubasa Ito, Naohisa Kishimoto, Jin Suminokura, Taira Hada, Yuka Otsuka, Kento Kato, Hiroaki Soyama, Masashi Takano

**Affiliations:** ^1^ Department of Obstetrics and Gynecology, National Defense Medical College Hospital, Tokorozawa, Saitama, Japan, ndmc.ac.jp; ^2^ Department of Obstetrics and Gynecology, Japan Self Defense Force Sapporo Hospital, Sapporo, Hokkaido, Japan

## Abstract

**Background and Aims:**

The optimal timing of cesarean delivery in women with placenta previa (PP) remains controversial because earlier delivery may reduce maternal risks, whereas later delivery may improve neonatal outcomes. This study aimed to evaluate whether delaying planned cesarean section (CS) until 38 gestational weeks was associated with improved neonatal outcomes without increasing maternal risks in women with PP who remain clinically stable.

**Methods:**

This historical control study included women with singleton pregnancies complicated by PP who underwent CS at a single tertiary center between 2015 and 2024. Planned CS was scheduled at 38^+0^ to 38^+6^ gestational weeks between 2022 and 2024 and at 36^+0^ to 37^+6^ gestational weeks between 2015 and 2022. Women who underwent emergency CS or developed obstetric complications, including antepartum hemorrhage or threatened preterm labor, were excluded from the primary analysis. Women who underwent planned CS at the scheduled gestational weeks were assigned to Group A (38‐week group) and Group B (36–37‐week group). Maternal and neonatal outcomes were compared between the groups.

**Results:**

Of the 204 eligible women, 29 were included in Group A and 81 in Group B. Maternal outcomes, including blood loss, operation time, and rates of severe complications, were comparable between the groups. In contrast, neonatal outcomes were improved in Group A, with higher birthweight and lower rates of neonatal hospitalization and hypoglycemia. Most obstetric complications occurred before 36 gestational weeks in both periods.

**Conclusion:**

Delaying planned CS until 38 gestational weeks in clinically stable women with PP was associated with improved neonatal outcomes without an apparent increase in maternal risks or emergency CS. These findings support further investigation of the optimal timing of delivery in women with PP.

## 1. Introduction

Placenta previa (PP) is a major cause of maternal and neonatal morbidity and mortality due to massive hemorrhage, with a reported incidence of 0.3%–1.2% [1–5].

The optimal timing of cesarean section (CS) for PP remains controversial because earlier delivery may reduce maternal risks, whereas later delivery may improve neonatal outcomes [1, 2, 4, 6]. Several guidelines recommend early delivery for PP due to concerns regarding antepartum hemorrhage (APH), preterm labor, and the increased likelihood of emergency CS as gestational age advances [4, 6–14]. The Royal College of Obstetricians and Gynecologists recommends delivery between 36^+0^ and 37^+0^ gestational weeks for uncomplicated PP and between 34^+0^ and 36^+6^ gestational weeks for women with a history of APH [4]. The American College of Obstetricians and Gynecologists recommends delivery between 37^+0^ and 37^+6^ gestational weeks in the absence of placenta accreta spectrum (PAS) [6], while Canadian guidelines recommend 37^+0^ to 37^+6^ gestational weeks for asymptomatic PP and 36^+0^ to 36^+6^ gestational weeks for symptomatic cases [7]. In contrast, Japanese guidelines recommend that elective CS for PP should be performed by 38 gestational weeks [4, 7, 8].

On the other hand, several studies in low‐risk pregnancies without obstetric complications have demonstrated that delaying elective CS until later gestational ages is associated with improved neonatal outcomes without increasing maternal morbidity [15–18]. However, these findings cannot be directly applied to PP because of the unique risk of sudden hemorrhage and emergency delivery in this population. Furthermore, the timing of obstetric complication onset in PP and its relationship with delivery timing have not been well characterized, leaving uncertainty regarding whether routine early delivery is always necessary in clinically stable patients.

At our institution, we have developed a standardized cesarean delivery technique using Bakri balloon tamponade, which has been associated with reduced perioperative bleeding [19]. This approach may allow for safer prolongation of pregnancy in selected women with PP.

Therefore, the aim of this study was to evaluate whether delaying planned CS until 38 gestational weeks is associated with improved neonatal outcomes without increasing maternal risks in women with PP who remain clinically stable.

## 2. Materials and Methods

This historical control study was conducted at the National Defense Medical College Hospital, Saitama, Japan. The aim of this study was to evaluate whether delaying planned cesarean delivery until 38 gestational weeks was associated with improved neonatal outcomes without increasing maternal risks in women with PP who remained clinically stable.

We reviewed electronic medical records of women with singleton pregnancies complicated by PP between January 2015 and December 2024. During 2022–2024, CS was initially planned at 38^+0^ to 38^+6^ gestational weeks for all women with PP, regardless of clinical presentation at the time of scheduling. During 2015–2022, CS was planned at 36^+0^ to 37^+6^ gestational weeks. In both periods, when patients developed obstetric complications such as APH or threatened preterm labor (TPL), clinical management was individualized. APH was defined as vaginal bleeding occurring before delivery. TPL was defined as uterine contractions with cervical shortening requiring inpatient management and administration of tocolytic agents. If these conditions were mild and could be controlled with conservative management, pregnancy was continued, and CS was rescheduled at 36–37 gestational weeks. In contrast, emergency CS was performed in cases of premature rupture of membranes (PROMs), uncontrollable uterine contractions, or persistent APH > 100 mL. Because APH and TPL are clinically important complications associated with an increased risk of emergency delivery and maternal hemorrhage [4, 6–8], women who developed these conditions and required individualized management were excluded from the primary analysis. Accordingly, the primary analysis focused on women with PP who remained clinically stable and underwent planned CS at the scheduled gestational age. Women who successfully underwent planned CS at 38^+0^ to 38^+6^ gestational weeks between 2022 and 2024 were assigned to Group A, whereas those who underwent planned CS at 36^+0^ to 37^+6^ gestational weeks between 2015 and 2022 were assigned to Group B. Women who required emergency CS and those who declined participation between 2022 and 2024 were also excluded from the primary analysis.

CS was performed using a standardized technique with Bakri balloon tamponade, as previously described [19]. The techniques were as follows: (1) a Bakri balloon prefilled with 50 mL of distilled water was inserted into the lower uterine segment via the uterine incision after placental separation, (2) the uterine incision site was closed, (3) the Bakri balloon was inflated with an additional 100–150 mL of water after the skin scar closure, and (4) vaginal gauze packing was applied to prevent balloon prolapse. Bakri balloon failure was defined as intraoperative or postoperative displacement of the balloon through the cervix into the vagina.

Baseline maternal characteristics included maternal age, parity, mode of conception, previous CS, clinical suspicion of PAS, type of PP, and placental location. Maternal outcomes included intraoperative blood loss, total blood loss within 24 h after CS, operation time, Bakri balloon failure, hemostatic hysterectomy, uterine artery embolization, and intensive care unit (ICU) admission. Neonatal outcomes included birthweight, Apgar scores at 1 and 5 minutes, umbilical artery pH, neonatal hospitalization, respiratory distress, use of mechanical ventilation, use of an incubator, hypoglycemia, and hyperbilirubinemia. Neonatal hospitalization was indicated when neonates were born preterm (< 37^+0^ gestational weeks), had low birthweight (< 2500 g), or required clinical management for conditions such as respiratory distress or hypoglycemia. These criteria remained unchanged throughout the study period. Respiratory distress was defined as failure to achieve adequate oxygen saturation after initial resuscitation or persistent signs of increased work of breathing requiring clinical intervention, including oxygen administration. Hypoglycemia was defined as a blood glucose level of < 50 mg/dL.

Continuous variables are presented as mean ± standard deviation or median (range), as appropriate, and categorical variables as number (%). Between‐group comparisons were performed using the Mann–Whitney *U* test for continuous variables and the chi‐square test or Fisher’s exact test for categorical variables, as appropriate. A *p* value < 0.05 was considered statistically significant. In addition, a multivariable regression model was used to identify factors associated with intraoperative blood loss, including delivery at 38 gestational weeks, history of previous CS, clinical suspicion of PAS, major PP, and anterior placenta as covariates. Statistical analyses were performed using JMP Pro Version 14 (SAS Institute Inc., Tokyo, Japan). Missing data were not observed for any variables included in the analysis. Because this study included all eligible cases during the study period, no a priori sample size calculation was performed, and the study protocol and data analysis plan were not prospectively registered. All analyses in this study were considered exploratory.

The study protocol was approved by the Institutional Review Board of the National Defense Medical College (approval date: May 28, 2019). Written informed consent was obtained from women scheduled for planned CS at 38^+0^ to 38^+6^ gestational weeks during the 2022–2024 period. For women included from the 2015 to 2022 period, comprehensive consent for research use of clinical data had been obtained at hospitalization. This study is reported in accordance with the STROBE guidelines, and the STROBE checklist is provided as Supporting File 1.

## 3. Results

**FIGURE 1:**
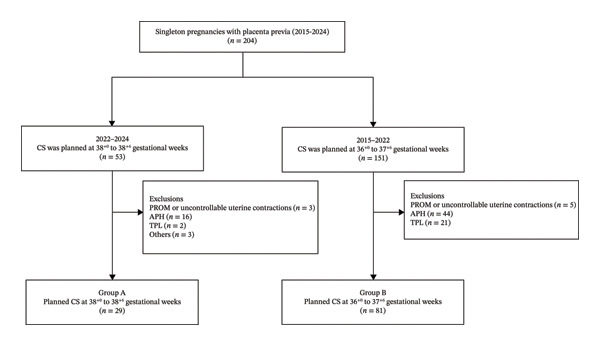
Study diagram. Women with singleton pregnancies complicated by placenta previa at our institution between 2015 and 2024 were initially identified. Planned CS was scheduled at 38^+0^ to 38^+6^ gestational weeks between 2022 and 2024 and at 36^+0^ to 37^+6^ gestational weeks between 2015 and 2022. Women who underwent emergency CS or developed controllable APH or TPL were excluded in both periods. Consequently, 29 women were included in Group A and 81 in Group B. Abbreviations: CS = cesarean section; APH = antepartum hemorrhage; TPL = threatened preterm labor.

A total of 204 women with singleton pregnancies complicated by PP between January 2015 and December 2024 were identified. Of the 53 women initially scheduled for planned CS at 38^+0^ to 38^+6^ gestational weeks (2022–2024), 24 were excluded from the primary analysis due to obstetric complications, including PROM or uncontrollable uterine contractions (*n* = 3), APH (*n* = 16), TPL (*n* = 2), and other reasons (*n* = 3). Similarly, of the 151 women initially scheduled for planned CS at 36^+0^ to 37^+6^ gestational weeks (2015–2022), 70 were excluded due to obstetric complications, including PROM or uncontrollable uterine contractions (*n* = 5), APH (*n* = 44), and TPL (*n* = 21). Finally, 29 women who underwent planned CS at 38^+0^ to 38^+6^ gestational weeks were assigned to Group A, and 81 women who underwent planned CS at 36^+0^ to 37^+6^ gestational weeks were assigned to Group B (Figure 1).

Baseline maternal characteristics were well balanced between the two groups (Table 1). There were no significant differences in maternal age, proportion of women aged ≥ 35 years, parity, mode of conception, or history of previous CS. In addition, there were no significant differences in the proportion of suspected PAS, type of PP, or placental location between the two groups.

**TABLE 1:** Baseline maternal characteristics.

Variables	Group A		Group B		*p* value
	*n* = 29		*n* = 81		
Age at CS, years	35.2 ± 5.6		33.9 ± 4.7		*p* = 0.23
Age ≥ 35 years, *n* (%)	18	(62.1%)	39	(48.2%)	*p* = 0.28
Parity, *n* (%)					*p* = 0.67
0	13	(44.8%)	41	(50.6%)	
≥ 1	16	(55.2%)	40	(49.4%)	
Mode of conception, *n* (%)					*p* = 0.18
Spontaneous pregnancy	21	(72.4%)	68	(84.0%)	
Other than spontaneous pregnancy	8	(27.6%)	13	(16.0%)	
History of previous CS, *n* (%)					*p* = 0.28
Yes	1	(3.5%)	10	(12.4%)	
No	28	(96.5%)	71	(87.6%)	
Clinical suspicion of PAS, *n* (%)					*p* = 0.99
Yes	1	(3.5%)	5	(6.2%)	
No	28	(96.5%)	76	(93.8%)	
Type of PP, *n* (%)					*p* = 0.62
Major PP	6	(20.7%)	22	(27.2%)	
Minor PP	23	(79.3%)	59	(72.8%)	
Placental location, *n* (%)					*p* = 0.99
Anterior placenta	2	(6.9%)	7	(8.6%)	
Posterior placenta	27	(93.1%)	74	(91.4%)	

*Note:* Major PP, total or partial placenta previa; Minor PP, marginal or low‐lying placenta previa.

Abbreviations: CS = cesarean section; PAS = placenta accreta spectrum; PP = placenta previa.

No statistically significant differences in maternal outcomes were observed between the two groups (Table 2). There were no significant differences in intraoperative blood loss, total blood loss within 24 h after cesarean delivery, or operation time. In the 2022–2024 period, APH occurred in 16 (30.2%) women and TPL in 2 (3.8%) women, whereas in the 2015–2022 period, APH occurred in 44 (29.1%) women and TPL in 21 (13.9%) women. Most cases of APH and TPL occurred before 36 gestational weeks in both periods. Furthermore, the incidence of Bakri balloon failure, uterine artery embolization, hemostatic hysterectomy, and ICU admission was similar between the two groups. Multivariable linear regression analysis showed that delivery at 38 gestational weeks was not independently associated with increased intraoperative blood loss. In contrast, major PP was independently associated with greater blood loss (Table 3).

**TABLE 2:** Maternal outcomes.

Variables	Group A		Group B		*p* value
	*n* = 29		*n* = 81		
Blood loss during CS, mL	895.2 ± 368.6		1004.1 ± 546.9		*p* = 0.62
Total blood loss until 24 h from CS, mL	1157.3 ± 442.1		1375.4 ± 1115.0		*p* = 0.94
Operation time, min	31.0 ± 8.9		34.0 ± 11.5		*p* = 0.21
Bakri balloon failure, *n* (%)	5	(17.2%)	10	(12.4%)	*p* = 0.54
Hemostatic hysterectomy, *n* (%)	0	(0.0%)	0	(0.0%)	*p* > 0.99
Uterine artery embolization, *n* (%)	0	(0.0%)	3	(3.7%)	*p* = 0.56
ICU admission, *n* (%)	0	(0.0%)	0	(0.0%)	*p* > 0.99

Abbreviations: CS = cesarean section; ICU = intensive care unit.

**TABLE 3:** Multivariable analysis of risk factors associated with intraoperative blood loss.

Variables	Hazard ratio	95% confidence interval	*p* value
Delivery at 38 gestational weeks	0.83	0.54–1.30	*p* = 0.40
History of previous CS	1.25	0.66–2.64	*p* = 0.51
Clinical suspicion of PAS	1.91	0.84–5.20	*p* = 0.13
Major PP	1.67	1.07–2.68	*p* = 0.02
Anterior placenta	1.55	0.73–3.70	*p* = 0.27

Abbreviations: CS = cesarean section; PAS = placenta accreta spectrum; PP = placenta previa.

Neonatal outcomes are shown in Table 4. Birthweight was significantly higher in Group A than in Group B (2920.0 ± 308.9 g vs. 2768.0 ± 304.3 g, *p* = 0.02). Apgar scores at 1 min were slightly higher in Group A, although the absolute difference was small (8.0 ± 1.1 vs. 8.0 ± 0.4, *p* = 0.03). The rate of neonatal hospitalization was significantly lower in Group A than in Group B (20.7% vs. 44.4%, *p* = 0.03). Among neonates who required hospitalization, respiratory distress was the most common reason. In addition, the incidence of hypoglycemia was significantly lower in Group A (3.5% vs. 19.8%, *p* = 0.04). There were no significant differences between the two groups in umbilical artery pH < 7.2, respiratory distress, use of mechanical ventilation, use of incubator, or hyperbilirubinemia.

**TABLE 4:** Neonatal outcomes.

Variables	Group A		Group B		*p* value
	*n* = 29		*n* = 81		
Birthweight, *g*	2920.0 ± 308.9		2768.0 ± 304.3		*p* = 0.02
Apgar score at 1 min	8.0 ± 1.1		8.0 ± 0.4		*p* = 0.03
Apgar score at 5 min	9.0 ± 0.5		8.8 ± 0.6		*p* = 0.09
Umbilical artery pH (< 7.2), *n* (%)	0	(0.0%)	1	(1.2%)	*p* = 0.99
Hospitalization, *n* (%)	6	(20.7%)	36	(44.4%)	*p* = 0.03
Respiratory distress, *n* (%)	3	(10.3%)	19	(23.5%)	*p* = 0.18
Use of mechanical ventilation, *n* (%)	1	(3.5%)	5	(6.2%)	*p* = 0.99
Use of incubator, *n* (%)	2	(6.9%)	18	(22.2%)	*p* = 0.09
Hypoglycemia, *n* (%)	1	(3.5%)	16	(19.8%)	*p* = 0.04
Hyperbilirubinemia, *n* (%)	0	(0.0%)	0	(0.0%)	*p* > 0.99

## 4. Discussion

In this historical control study, delaying planned cesarean delivery until 38 gestational weeks in women with PP who remained clinically stable was associated with improved neonatal outcomes, while no statistically significant differences in maternal outcomes were observed. Specifically, birthweight was higher, and the rates of neonatal hospitalization and hypoglycemia were lower among women scheduled for planned cesarean delivery at 38 gestational weeks, whereas maternal outcomes and the incidence of emergency cesarean delivery remained comparable between the two groups.

Most cases of APH and TPL occurred before 36 gestational weeks in both periods, consistent with previous reports showing that these are common complications in women with PP [20–27].

Current clinical practice and guidelines generally recommend delivery at 36–37 gestational weeks in PP due to concerns regarding maternal hemorrhage and emergency cesarean delivery [4, 6–14]. However, these recommendations are largely based on the potential risk of sudden bleeding and may not fully reflect the actual clinical course of the disease. In light of the present findings, routine early delivery at 36–37 gestational weeks may not always be necessary in clinically stable patients.

The observed differences in neonatal outcomes between the two groups may be clinically meaningful. Although the overall incidence of respiratory distress did not differ significantly between the groups, the higher birthweight and lower incidence of hypoglycemia observed in the 38‐week group may have contributed to the lower rate of neonatal hospitalization. In contrast, the difference in Apgar score at 1 min, despite statistical significance, was small and of uncertain clinical relevance. These findings are consistent with previous reports demonstrating improved neonatal outcomes with increasing gestational age, even within the early‐term period [15–18]. Given that PP itself is associated with increased neonatal morbidity and mortality [28], optimizing the timing of delivery in this population is particularly important.

No statistically significant differences in maternal outcomes were observed between the two groups, and no increase in hemorrhage‐related outcomes or emergency cesarean delivery was observed in the 38‐week group. However, the relatively small number of patients in the 38‐week group may have limited the statistical power to detect differences in maternal outcomes. Therefore, further discussion of this issue is warranted. When planned cesarean delivery was scheduled at 38^+0^ to 38^+6^ gestational weeks, a certain proportion of women developed obstetrical complications such as APH or TPL and therefore required earlier delivery. Accordingly, careful surveillance and timely intervention were necessary for these women. Notably, although birthweight was higher in the 38‐week group, this did not translate into increased intraoperative blood loss. This finding contrasts with previous reports demonstrating an association between higher birthweight and greater intraoperative hemorrhage in women with PP [29]. Furthermore, exploratory multivariable analysis demonstrated that delivery at 38 gestational weeks was not independently associated with greater blood loss, whereas major PP was associated with increased hemorrhage. The relatively high rate of Bakri balloon failure observed in both groups may be attributable to cervical dilation or ripening, which may have made it difficult for the balloon to remain in place at the internal cervical os.

Several limitations of this study should be acknowledged when interpreting the findings. First, this was a single‐center historical control study, which may limit generalizability. In addition, the exclusion of women who required emergency cesarean delivery or developed controllable APH or TPL may have introduced selection bias, particularly in the 38‐week group. Because these women likely represented a higher‐risk population among women with PP, the maternal and neonatal risks observed in this study may not fully represent those of the overall PP population. Therefore, our findings should be interpreted as reflecting outcomes among carefully selected women who remained candidates for planned cesarean delivery. Furthermore, because this study compared patients from different time periods, temporal changes in obstetric, anesthetic, neonatal, and perioperative management may have influenced the observed outcomes despite the use of standardized institutional protocols. In addition, potential confounding factors associated with neonatal outcomes could not be fully evaluated in this study. Clinically relevant factors such as fetal growth restriction, hypertensive disorders of pregnancy, and maternal diabetes were not uniformly available in this retrospective dataset. Additional studies with larger numbers of patients are warranted to clarify the influence of these factors on neonatal outcomes. Second, although exploratory multivariable analysis was performed for intraoperative blood loss, residual confounding could not be fully excluded because of the limited sample size and retrospective study design. Taken together, these limitations should be considered when interpreting the findings, and further studies with larger sample sizes and more comprehensive analytical methods would be valuable.

Despite these limitations, this study has several strengths. The management strategy was clearly defined and consistently applied across the study periods, and detailed information regarding the timing and clinical course of obstetric complications was available. This enabled us to evaluate not only outcomes but also the clinical context in which these outcomes occurred. As a result, the present study provides clinically relevant insight into the feasibility and safety of delaying planned cesarean delivery in carefully selected women with PP.

## 5. Conclusions

In women with PP who remained clinically stable, delaying planned cesarean delivery until 38 gestational weeks was associated with improved neonatal outcomes without an apparent increase in maternal risks or the incidence of emergency cesarean delivery. Further studies are warranted to determine the optimal timing of delivery in women with PP.

## Author Contributions

Minori Takada had full access to all of the data in this study and takes complete responsibility for the integrity of the data and the accuracy of the data analysis.

## Funding

This research received no specific grant from any funding agency in the public, commercial, or not‐for‐profit sectors.

## Disclosure

The study was conducted as part of the authors’ routine clinical practice at the National Defense Medical College Hospital. All authors have read and approved the final version of the manuscript. Minori Takada affirms that this manuscript is an honest, accurate, and transparent account of the study being reported; that no important aspects of the study have been omitted; and that any discrepancies from the study as planned have been explained.

## Conflicts of Interest

The authors declare no conflicts of interest.

## Supporting Information

Additional supporting information can be found online in the Supporting Information section.

## Supporting information


**Supporting Information** The STROBE checklist for this study is provided as Supporting File 1.

## Data Availability

The authors confirm that the data supporting the findings of this study are available within the article.
