# Supplementary material for: Cesarean Section at 38 Gestational Weeks for Placenta Previa was Associated With Improved Neonatal Outcomes Without an Apparent Increase in Maternal Risks: A Historical Control Study
Source: Obstet Gynecol Int. 2026 Jul 24;2026:1616243. doi: 10.1155/ogi/1616243 (PMC13397474; doi:10.1155/ogi/1616243)
Supplement: Supplementary file 1 — Supporting Information The STROBE checklist for this study is provided as Supporting File 1. [file OGI-2026-1616243-s001.docx]

STROBE Statement—checklist of items that should be included in reports of observational studies

|  | | Item No | Recommendation |
| --- | --- | --- | --- |
| **Title and abstract** | | 1 | (*a*) Indicate the study’s design with a commonly used term in the title or the abstract  Historical control study as stated in the Title on page1, Abstract on page 2 and Materials and Methods on page 4. |
|  |  |  | (*b*) Provide in the abstract an informative and balanced summary of what was done and what was found  Provided in the Abstract on page 2. |
| Introduction | | | |
| Background/rationale | | 2 | Explain the scientific background and rationale for the investigation being reported  Included in the Introduction on pages 3. |
| Objectives | | 3 | State specific objectives, including any prespecified hypotheses  Included in the Introduction on page 3. |
| Methods | | | |
| Study design | | 4 | Present key elements of study design early in the paper  Included in the Materials and Methods on page 4. |
| Setting | | 5 | Describe the setting, locations, and relevant dates, including periods of recruitment, exposure, follow-up, and data collection  Included in the Materials and Methods on pages 4–5. |
| Participants | | 6 | (*a*) *Cohort study*—Give the eligibility criteria, and the sources and methods of selection of participants. Describe methods of follow-up  Included in the Materials and Methods on pages 4–5.  *Case-control study*—Give the eligibility criteria, and the sources and methods of case ascertainment and control selection. Give the rationale for the choice of cases and controls  *Cross-sectional study*—Give the eligibility criteria, and the sources and methods of selection of participants |
|  |  |  | (*b*) *Cohort study*—For matched studies, give matching criteria and number of exposed and unexposed  Not applicable.  *Case-control study*—For matched studies, give matching criteria and the number of controls per case |
| Variables | | 7 | Clearly define all outcomes, exposures, predictors, potential confounders, and effect modifiers. Give diagnostic criteria, if applicable  Included in the Materials and Methods on pages 4–5. |
| Data sources/ measurement | | 8* | For each variable of interest, give sources of data and details of methods of assessment (measurement). Describe comparability of assessment methods if there is more than one group  Included in the Materials and Methods on pages 4–5. Data were obtained from electronic medical records. |
| Bias | | 9 | Describe any efforts to address potential sources of bias  Addressed in the limitations section of the Discussion on pages 8–9. |
| Study size | | 10 | Explain how the study size was arrived at  Included in the Materials and Methods on page 5. |
| Quantitative variables | | 11 | Explain how quantitative variables were handled in the analyses. If applicable, describe which groupings were chosen and why  Included in the Materials and Methods on page 5. |
| Statistical methods | | 12 | (*a*) Describe all statistical methods, including those used to control for confounding  Included in the Materials and Methods on page 5. |
|  |  |  | (*b*) Describe any methods used to examine subgroups and interactions  Not applicable. |
|  |  |  | (*c*) Explain how missing data were addressed  Included in the Materials and Methods on page 5. |
|  |  |  | (*d*) *Cohort study*—If applicable, explain how loss to follow-up was addressed  Not applicable.  *Case-control study*—If applicable, explain how matching of cases and controls was addressed  *Cross-sectional study*—If applicable, describe analytical methods taking account of sampling strategy |
|  |  |  | (*e*) Describe any sensitivity analyses  Exploratory multivariable analysis is described in the Materials and Methods on page 5 and Results on page 6. |
| Results | | | |
| Participants | 13* | (a) Report numbers of individuals at each stage of study—eg numbers potentially eligible, examined for eligibility, confirmed eligible, included in the study, completing follow-up, and analysed  Included in the Results on page 6 and Figure 1. | |
|  |  | (b) Give reasons for non-participation at each stage  Included in the Results on page 6 and Figure 1. | |
|  |  | (c) Consider use of a flow diagram  Provided as Figure 1. | |
| Descriptive data | 14* | (a) Give characteristics of study participants (eg demographic, clinical, social) and information on exposures and potential confounders  Included in the Results on page 6 and Table 1. | |
|  |  | (b) Indicate number of participants with missing data for each variable of interest  Included in the Materials and Methods on page 5. | |
|  |  | (c) *Cohort study*—Summarise follow-up time (eg, average and total amount)  Not applicable. | |
| Outcome data | 15* | *Cohort study*—Report numbers of outcome events or summary measures over time  Included in the Results on pages 6–7 and Tables 2–4. | |
|  |  | *Case-control study—*Report numbers in each exposure category, or summary measures of exposure | |
|  |  | *Cross-sectional study—*Report numbers of outcome events or summary measures | |
| Main results | 16 | (*a*) Give unadjusted estimates and, if applicable, confounder-adjusted estimates and their precision (eg, 95% confidence interval). Make clear which confounders were adjusted for and why they were included  Included in Tables 1, 2, and 4 (unadjusted analyses) and Table 3 (multivariable adjusted analysis with 95% confidence intervals). | |
|  |  | (*b*) Report category boundaries when continuous variables were categorized  Included in the Materials and Methods on page 5. | |
|  |  | (*c*) If relevant, consider translating estimates of relative risk into absolute risk for a meaningful time period.  Not applicable. | |
| Other analyses | 17 | Report other analyses done—eg analyses of subgroups and interactions, and sensitivity analyses  Exploratory multivariable analysis is reported in Table 3 and described in the Results section. | |
| Discussion | | | |
| Key results | 18 | Summarise key results with reference to study objectives  Included in the Discussion on pages 8–9. | |
| Limitations | 19 | Discuss limitations of the study, taking into account sources of potential bias or imprecision. Discuss both direction and magnitude of any potential bias  Included in the Discussion on pages 8–9. | |
| Interpretation | 20 | Give a cautious overall interpretation of results considering objectives, limitations, multiplicity of analyses, results from similar studies, and other relevant evidence  Included in the Discussion on pages 8–9. | |
| Generalisability | 21 | Discuss the generalisability (external validity) of the study results  Included in the Discussion on page 9. | |
| Other information | | | |
| Funding | 22 | Give the source of funding and the role of the funders for the present study and, if applicable, for the original study on which the present article is based  Provided in the Funding Statement on page 11. | |
